# Baculum morphology predicts reproductive success of male house mice under sexual selection

**DOI:** 10.1186/1741-7007-11-66

**Published:** 2013-06-26

**Authors:** Paula Stockley, Steven A Ramm, Amy L Sherborne, Michael D F Thom, Steve Paterson, Jane L Hurst

**Affiliations:** 1 Mammalian Behaviour & Evolution Group, Department of Evolution, Ecology and Behaviour, University of Liverpool, Leahurst Campus, Chester High Road, Neston CH64 7TE, UK; 2 Department of Evolutionary Biology, Bielefeld University, Morgenbreede 45, Bielefeld 33615, Germany; 3 Department of Evolution, Ecology and Behaviour, University of Liverpool, Crown Street, Liverpool L69 7ZB, UK; 4 Section of Cancer Genetics, Institute of Cancer Research, Cotswold Road, Sutton, Surrey SM2 5NG, UK; 5 Department of Biology, University of York, Wentworth Way, Heslington, York Y010 5DD, UK

**Keywords:** Baculum, Cryptic female choice, Genital evolution, Os penis, Penile morphology, Postcopulatory sexual selection, Sperm competition

## Abstract

**Background:**

Diversity in penile morphology is characterised by extraordinary variation in the size and shape of the baculum (penis bone) found in many mammals. Although functionally enigmatic, diversity in baculum form is hypothesised to result from sexual selection. According to this hypothesis, the baculum should influence the outcome of reproductive competition among males within promiscuous mating systems. However, a test of this key prediction is currently lacking.

**Results:**

Here we show that baculum size explains significant variation in the reproductive success of male house mice under competitive conditions. After controlling for body size and other reproductive traits, the width (but not length) of the house mouse baculum predicts both the mean number of offspring sired per litter and total number of offspring sired.

**Conclusions:**

By providing the first evidence linking baculum morphology to male reproductive success, our results support the hypothesis that evolutionary diversity in baculum form is driven by sexual selection.

## Background

**Figure 1:**
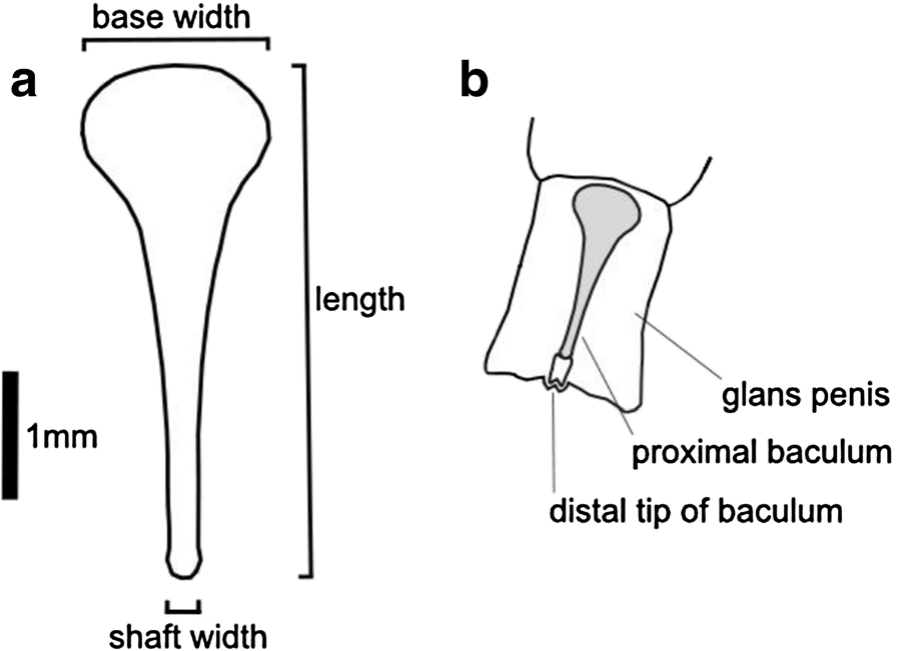
**Baculum morphology in the house mouse.** (**a**) Line drawing of the main proximal bone of the baculum (dorsal view), indicating the three dimensions (maximum length, maximum base width, minimum shaft width) used in this study. (**b**) Line drawing of the same bone (shaded) *in situ*, illustrating the baculum’s position within the glans penis. Redrawn from [19].

Diversity in intromittent genitalia (so-called ‘genitalic extravagance’ [1]) is a widespread evolutionary phenomenon that is hypothesised to result from postcopulatory sexual selection [1-4]. Consistent with this general pattern, the mammalian baculum (os penis) is characterised by extreme anatomical variation across diverse mammalian species (including rodents, bats, primates and carnivores) [5-9]. However, current evidence of sexual selection on the baculum is limited, based on comparative and indirect studies of baculum size [10-18]. Here, we test if baculum size in wild house mice (*Mus musculus domesticus*) (Figure 1) predicts male reproductive success under competitive conditions.

The house mouse is a model species for studies of reproductive behaviour [20], and regularly mates promiscuously in the wild [21]. To quantify key components of male reproductive success under competitive conditions we used four discrete experimental populations of house mice in very large outdoor enclosures, providing natural and controlled conditions within which all animals could be sampled (see Methods). Our analyses include expected effects of dominance and body size on male mating and fertilisation success [22] and test for the effects of additional traits predicted to influence the outcome of postcopulatory sexual selection (testis size, accessory reproductive gland size) [23,24].

## Results

### Variation in male reproductive success

A total of 241 surviving offspring were sired by 24 focal males in the outdoor enclosures. The number of offspring sired per male ranged from 0 to 27 (mean ± s.e. = 10.04 ± 1.90). Overall, 67% of litters were multiply sired. Focal males that achieved at least one copulation from which offspring were identified (n = 21) sired an average of 2.21 ± 0.21 (s.e.) offspring per litter (indicative of success in postcopulatory sexual selection under high levels of multiple mating). Variation in male reproductive success and high levels of multiple paternity reveal evidence of intense competition between males in our experimental populations. In the following sections, we analyse male traits contributing to variation in reproductive success. Mean measurements of morphological traits [see Additional file 1: Table S1] and results of Pearson correlation tests between traits [see Additional file 1: Table S2] are provided as supplementary information.

### Baculum size

Baculum size significantly predicted the reproductive success of male house mice after controlling for effects of body mass and other reproductive traits (Table 1, Table 2; see Additional file 1: Tables S3 and S4 for results using principal component analysis). Specifically, baculum shaft width significantly predicted the number of offspring sired by focal males in our study populations (Table 1). Baculum shaft width and base width were also significant predictors of the mean number of offspring sired per litter (Table 2), consistent with an influence on differential reproductive success under postcopulatory sexual selection. By contrast, baculum length had no significant influence on measures of male reproductive success independent of body mass and other reproductive traits.

**Table 1:** Number of offspring sired by male house mice

**Fixed effects**	**Coefficient (SE)**	**z-value**	***P*-value**	Δ**AIC**	**Random effects**	**Variance (SD)**
Model for total number of offspring sired						
Intercept	−26.14 (5.42)	−4.83	<0.001	-	Sibling group	0.47 (0.69)
Body mass	4.52 (1.55)	2.91	0.004	-	Population	0.37 (0.61)
Testes mass	19.43 (6.50)	2.99	0.003	5.82		
Seminal vesicles mass	8.53 (3.35)	2.54	0.011	3.71		
Baculum shaft width	32.24 (5.32)	6.06	<0.001	45.04		

Generalized linear mixed model to investigate morphological traits influencing the reproductive success of male house mice, showing the best model for the total number of offspring sired. Data for morphological traits were log transformed prior to analysis. Body mass was retained in the model as a covariate. Population and sibling group were included as random effects (see text for further details). ΔAIC = the change in the AIC if the single term is dropped. Number of observations = 22, sibling groups = 11, populations = 4.

**Table 2:** Paternity success of male house mice

**Fixed effects**	**Estimate (SE)**	***t*value**	***P*-value**	Δ**AIC**	**Random effects**	**Variance (SD)**
Model for average number of offspring per litter						
Intercept	−22.51 (3.61)	−6.23	-	-	Sibling group	1.80 (1.34)
Body mass	6.07 (1.13)	5.37	<0.001	-	Population	0.10 (0.31)
Baculum shaft width	9.35 (4.25)	2.20	0.013	4.19		
Baculum base width	8.50 (2.06)	4.13	0.020	3.42		

Linear mixed model to investigate morphological traits influencing the reproductive success of male house mice, showing the best model for the average number of offspring sired per litter. Data for morphological traits were log transformed prior to analysis. Body mass was retained in the model as a covariate. Population and sibling group were included as random effects (see text for further details). ΔAIC = the change in the AIC if the single term is dropped. Number of observations = 19, sibling groups = 11, populations = 4.

### Other traits linked to male reproductive success

Body mass, testis mass and the mass of the seminal vesicles (accessory reproductive glands) were also significant predictors of the total number of offspring sired by male house mice under competitive conditions (Table 1). By contrast the mass of the preputial glands, which are used in scent marking and reliably linked to male dominance [25], did not predict male reproductive success.

## Discussion

Our findings reveal that baculum morphology (specifically width) predicts the reproductive success of male house mice independently of other key traits linked to competitive ability (body mass, testis mass, seminal vesicle mass, preputial gland mass). Perhaps the most likely explanation for this finding is that a wider baculum facilitates increased stimulation of the female reproductive tract. In house mice there is ample opportunity for such stimulation to occur during a prolonged copulatory sequence consisting of multiple intromissions with thrusting [20]. Copulatory stimulation is known to trigger complex neuroendrocrine responses in female mammals, including house mice, and may assist in sperm transport as well as maintenance of pregnancy [26-28]. A wider baculum might also assist in dislodging copulatory plugs left by rival males during pre-ejaculatory intromissions, or in achieving intromission [7-9]. However, the precise mechanisms by which the baculum might promote differential fertilisation success remain to be determined.

Our findings support previous suggestions that baculum size is subject to sexual selection. For example, it has been hypothesised that positive allometry and heightened coefficients of phenotypic variation may be indicative of sexual selection on genitalic and other traits [29] (but see [30,31]). Accordingly, several studies of rodents and carnivores report evidence of positive allometry and high variability in measures of baculum length or width [12-18]. In addition, comparative analyses indicate that baculum size may be subject to sexual selection in rodents and carnivores, with increased baculum length favoured under high levels of sperm competition [10]. In the present study however, we find baculum width to be a more important predictor of reproductive success for male house mice under conditions of intense postcopulatory sexual selection, which is consistent with relatively high intraspecific variation [16,17] and positive allometry [17,18] in this aspect of penile morphology.

Significant effects of body mass and testes mass on male reproductive success are to be expected under pre- and postcopulatory sexual selection. Large males may gain a reproductive advantage through contest competition or female choice [22,26]. Similarly, large testes are expected to confer an advantage in sperm competition, by facilitating increased numbers of sperm per ejaculate and more frequent ejaculation [23,32-35]. Here we also report a significant influence of seminal vesicles on male reproductive success. Our findings provide the first such evidence under competitive conditions, consistent with the hypothesis that a larger copulatory plug (the major product of seminal vesicle secretions in this species [36]) may be more effective in delaying female remating and/or promoting sperm transport [24,37,38].

Measuring reproductive success under natural conditions is challenging [22] and particularly so in relation to genital morphology. Importantly, potential confounding effects normally present in natural populations were either controlled within our experimental enclosures (for example, variation in age, previous experience, genetic origin and pedigree of focal animals) or taken into account in our analyses (body size, dominance, testis size, accessory gland size, population, relatedness of focal animals). The capture of all surviving offspring produced over sequential reproductive events (not normally possible for free-living populations due to dispersal and predation events), further strengthens our conclusions. Nonetheless, future experimental studies would be beneficial to explore our findings further under controlled laboratory conditions, ideally involving a creative approach to manipulate baculum size independently of other reproductive traits (compare to [39]).

## Conclusions

In conclusion, our finding that penile morphology predicts key components of male reproductive success under natural and controlled conditions provides a significant advance in the study of genital evolution, complementing increasing numbers of comparative and laboratory-based studies in diverse taxa [2,40]. More specifically our results support the hypothesis that baculum diversity is driven by sexual selection, with broad implications for understanding patterns of morphological diversification and speciation [1,2]. In particular, a focus on divergence in baculum morphology between closely related species or diverging populations is likely to advance understanding of genital evolution and speciation processes, as is increasingly being recognised in arthropod taxa [41,42]. Further work is now required to determine the mechanisms by which the baculum interacts with female reproductive traits to affect the outcome of postcopulatory sexual selection, driving the extraordinarily divergent evolution of penile morphology.

## Methods

### Ethical statement

This research adhered to the Association for the Study of Animal Behaviour/Animal Behaviour Society Guidelines for the Use of Animals in Research, was approved by the University of Liverpool’s Animal Welfare Committee, and carried out under a UK Home Office Licence.

### Subjects

Wild house mice (*M. musculus domesticus*) were captive-bred F1-F3 animals from a large outbred colony originating from five different populations in the northwest of England, UK. Animals were housed in standard rodent cages (40 × 23.5 × 12.5 cm, North Kent Plastic Cages Ltd, UK), with Corn Cob Absorb 10/14 substrate and paper wool bedding material, and *ad libitum* access to food (LabDiet 5002 Certified Rodent Diet, Purina Mills, St Louis, MO, USA) and water. Controlled environmental conditions were maintained at 20 to 21°C, 45% to 65% relative humidity, and a reversed 12–12 hour light cycle (lights off at 0800).

Four breeding groups were established to produce offspring for release into outdoor enclosures. Breeding groups contained two sets of three unrelated females, with each set of females housed for 14 days with an unrelated male (unrelated animals shared no parents or grandparents). This breeding plan was designed to simulate a naturalistic situation in which dominant males could sire offspring with several unrelated females resulting in discrete local populations [43]. At weaning (four weeks), offspring were given subcutaneous radio-frequency identification tags (RFID), and a small tissue sample was taken from the tip of the tail (1 to 2 mm) under general anaesthesia (halothane) for genotyping. Animals were then separated into single sex sibling groups and housed under controlled environmental conditions (see above). Social and environmental experience was thus carefully controlled with no opportunity for mating prior to release. All resulting offspring from each breeding group were released together simultaneously as founders into four separate outdoor enclosures, at age 48 to 65 days. In total, 48 male and 33 female founder mice were released to create four separate breeding populations (A: 7 males, 6 females; B: 18 males, 12 females; C: 12 males, 7 females; D: 11 males, 8 females). To investigate relationships with reproductive success, male morphological traits were measured for a sample of 24 focal males selected randomly from the four populations (A: 2/7 males, B: 11/18 males, C: 6/12 males, D: 5/7 males, see below).

### Population enclosures

Outdoor enclosures (25×10 m) contained long grass as ground cover and sufficient space for all released animals to establish territories. Thirty nest boxes were provided within each enclosure and ten concrete block shelters (45 × 45 × 35 cm) were added after 12 weeks for additional shelter. Ten food and water stations, spaced evenly around the outer walls of each enclosure, provided *ad libitum* food (Lab Diet 5002 Certified Rodent Diet) and water. Sheet-aluzinc walls (1.3 m high with concrete foundations) prevented escape or contact between populations, and wire mesh upper walls and roof prevented predation. Mice were left to breed undisturbed for 15 to 19 weeks after release, allowing sufficient time for females to rear up to three litters to independence. Each of the four populations was then removed from the enclosures by live trapping. Sex, weight and age class (adult, sub-adult, juvenile) were recorded for all captured animals. Individually tagged ‘founder’ animals were identified and returned to the laboratory, with males housed individually under controlled environmental conditions (cages 48.5 × 11.5 × 12 cm, see above). Descendants of the founder animals were culled humanely under halothane anaesthetic and tail snips were taken for genotyping to establish parentage.

### Parentage and mating assignment

Reproductive success of founder males was quantified by assigning parentage to F2 offspring (distinguished from potential F3 descendants on the basis of their weight, sex and date of capture) and is described in detail elsewhere [43]. Briefly, all F1 and F2 individuals were genotyped for a set of 24 unlinked microsatellite markers with individuals assigned to parent pairs using maximum-likelihood methods implemented in CERVUS v3.0 [44] and further validated with reference to major histocompatibility complex (MHC) and major urinary protein (MUP) genotyping [43].

Offspring were assigned to litters for each founder female based on sex-specific growth curves. Methods are described in detail elsewhere [43]. Briefly, for each female, we plotted offspring weight against capture date and used the sex-specific growth curves from captive F2 animals together with paternity to assign offspring to separate litters. We took a conservative approach and only assigned offspring from the same sire to separate copulations if they were very unlikely to come from the same litter (based on their body mass and a maximum litter size of 9 for wild house mice). For focal males that did not sire offspring in the outdoor enclosures, subsequent pairings with females from the same population confirmed normal fertility. This was achieved on recapture by pairing each focal male with a (non-sibling) female in MB1 cages (45 × 28 × 13 cm) under standard laboratory conditions (as described above), and monitoring pairs for litter production.

### Measurement of baculum and other male reproductive traits

To investigate relationships between male genitalic traits and reproductive success, focal male mice (see above) were culled humanely under halothane anaesthetic. Bacula were prepared post-mortem following dissection and storage of penises at −20°C. On defrosting, bacula were thoroughly cleaned of surrounding tissue using a combination of dissection under a microscope at 20× magnification and repeated soaking in 0.05 g ml-1 KOH (24 hours × 2), and stored in 70% ethanol [17]. The baculum in house mice consists of a large proximal bone (representing the main base and shaft of the baculum) and a much smaller distal bone at its tip (Figure 1, see also [45]). In this study the small distal bone was removed during the cleaning process and measurements were made of the large proximal bone (Figure 1). This bone is relatively simple in form. Consistent with previous functional analyses of the baculum [10-18], we have therefore focused our analysis on measurements of baculum length and width - two major, independent axes of morphological variation [see Additional file 1: Tables S1 and S2]. A digital image of each bone was obtained using a flatbed scanner (CanoScanLiDE 30, Canon Inc.) at a resolution of 1,200 dpi, using a solid black background to create contrast during scanning. Images were processed using Image J software (version 1.38×, [46]) with measurements obtained digitally using the Measure, Threshold and Analyse Particles tools. Following inversion, images were converted to 32-bit and rotated if necessary to align the image on a vertical axis before setting the scale. For each baculum, we recorded maximum length (baculum length), maximum width of the base (baculum base width), and width of the shaft at its narrowest point (baculum shaft width) (Figure 1). The latter two measurements are positively correlated, whereas length and width varied independently in our dataset and so we analysed these aspects of baculum variation separately [see Additional file 1: Table S2]. Linear measurements were made to the nearest 0.01 mm (length and base width) or 0.001 mm (shaft width). Sample sizes for the different measurements vary because some bacula were damaged during processing.

Also measured at dissection were testes mass, seminal vesicles mass, and preputial glands mass (each paired, to the nearest 0.001 g). Mean body mass was calculated as an average of pre-release, post-capture and post-mortem body mass (to the nearest 0.01 g).

### Statistical analysis

To investigate morphological traits influencing the total number of offspring produced by the subject males, we used generalized linear mixed models (GLMMs) with a logarithm link function and Poisson distribution, fitted using the Laplace approximation to restricted maximum likelihood estimation (lmer procedure in the lme4 R package [47]). To investigate traits influencing the average number of offspring sired per litter we used linear mixed effect models (LMEs), fitted by maximum likelihood using the lme4 package in R. Significance was assessed by comparison of the model with and without the variable of interest included, using likelihood ratio tests. For both approaches (GLMMs, LMEs), population and focal male sibling group were included as random effects, to control for relatedness of subjects and shared environmental and social conditions within the four outdoor enclosures. All morphological traits were log transformed prior to analysis to ensure normality.

In the first stage of analysis to investigate morphological traits influencing male reproductive success, we constructed separate models for each trait of interest (baculum length, shaft width and base width, testis mass, seminal vesicle mass, preputial gland mass), with body mass as a covariate. Traits with significant influence were then combined for each measure of male reproductive success to fit the best model in each case, with body mass again retained as a covariate. Results of analyses for individual traits and combined models are provided in Additional file 1: Tables S5 and S6. Preliminary analyses revealed no significant influence of variation in sex ratio of founder animals on measures of male reproductive success; hence sex ratio was not included in further analyses. Results of analyses with population as a fixed effect are provided in Additional file 1: Tables S7 and S8.

## Abbreviations

GLMMs: Generalized linear mixed models; LMEs: Linear mixed effect models; AIC: Akaike information criterion.

## Competing interests

The authors declare that they have no competing interests.

## Authors’ contributions

PS and JLH conceived and designed the study, all authors collected data and PS, JLH and SP analysed data. PS drafted the manuscript with help from SR and all authors contributed to revisions. JLH, PS and SP gained funding. All authors read and approved the final manuscript.

## Supplementary Material

Additional file 1: Table S1 Provides descriptive information regarding measurements of male reproductive traits (sample size, mean, standard error, minimum and maximum values). **Table S2.** Provides Pearson correlation coefficients calculated between measures of morphological traits used in main analyses. **Table S3.** Shows results of generalized linear mixed model to investigate morphological traits influencing male reproductive success, using results of principal components analysis for baculum size measurements. **Table S4.** Shows results of linear mixed model to investigate morphological traits influencing male paternity success, using results of principal components analysis for baculum size measurements. **Table S5.** Shows results of generalized linear mixed models to investigate morphological traits influencing male reproductive success, including analysis of individual morphological traits, and combined models for significant traits. **Table S6.** Shows results of linear mixed models to investigate morphological traits influencing male paternity success, including analysis of individual morphological traits, and combined models for significant traits. **Table S7.** Shows results of generalized linear mixed model to investigate morphological traits influencing male reproductive success, with population as a fixed effect. **Table S8.** Shows results of linear mixed model to investigate morphological traits influencing male paternity success, with population as a fixed effect. Additional file methods-Describes principal components analysis presented in Additional file 1: Tables S3 and S4. Click here for file
