# Supplementary material for: Baculum morphology predicts reproductive success of male house mice under sexual selection
Source: BMC Biol. 2013 Jun 26;11:66. doi: 10.1186/1741-7007-11-66 (PMC3693876; doi:10.1186/1741-7007-11-66)
Supplement: Additional file 1: Table S1 — Provides descriptive information regarding measurements of male reproductive traits (sample size, mean, standard error, minimum and maximum values). Table S2. Provides Pearson correlation coefficients calculated between measures of morphological traits used in main analyses. Table S3. Shows results of generalized linear mixed model to investigate morphological traits influencing male reproductive success, using results of principal components analysis for baculum size measurements. Table S4. Shows results of linear mixed model to investigate morphological traits influencing male paternity success, using results of principal components analysis for baculum size measurements. Table S5. Shows results of generalized linear mixed models to investigate morphological traits influencing male reproductive success, including analysis of individual morphological traits, and combined models for significant traits. Table S6. Shows results of linear mixed models to investigate morphological traits influencing male paternity success, including analysis of individual morphological traits, and combined models for significant traits. Table S7. Shows results of generalized linear mixed model to investigate morphological traits influencing male reproductive success, with population as a fixed effect. Table S8. Shows results of linear mixed model to investigate morphological traits influencing male paternity success, with population as a fixed effect. Additional file methods-Describes principal components analysis presented in Additional file 1: Tables S3 and S4. [file 1741-7007-11-66-S1.docx]

**Additional file**

**Baculum morphology predicts reproductive success of male house mice under sexual selection**

**Paula Stockley, Steven A. Ramm, Amy L. Sherborne, Michael D.F. Thom,**

**Steve Paterson & Jane L. Hurst**

**Additional file 1**

**Additional file 1: Table S1** Mean, standard error (SE), minimum and maximum values for morphological reproductive traits of focal male house mice. N (sample size) varies due to missing data for certain morphological traits. Data for testes, seminal vesicles and preputial glands are for paired organ masses.

| **Male trait** | **N** | **Minimum** | **Maximum** | **Mean** | **SE** |
| --- | --- | --- | --- | --- | --- |
| Body mass (g) | 24 | 18.46 | 25.65 | 21.83 | 0.38 |
| Testes mass (g) | 24 | 0.14 | 0.23 | 0.19 | 0.005 |
| Seminal vesicles mass (g) | 24 | 0.14 | 0.35 | 0.22 | 0.01 |
| Preputial glands mass (g) | 24 | 0.04 | 0.14 | 0.08 | 0.005 |
| Baculum length (mm) | 22 | 3.76 | 4.59 | 4.23 | 0.05 |
| Baculum shaft width (mm) | 22 | 0.25 | 0.42 | 0.33 | 0.01 |
| Baculum base width (mm) | 23 | 1.31 | 1.76 | 1.50 | 0.03 |

**Additional file 1: Table S2** Pearson correlation coefficients calculated between measures of morphological traits for focal male house mice. Data were log transformed prior to analysis. Figures in parentheses are the number of animals included in each analysis. Sample sizes vary due to missing data for certain morphological traits. ** P <0.01; * P = 0.05.

| **Male trait** | BM | TM | SVM | PGM | BL | BBW |
| --- | --- | --- | --- | --- | --- | --- |
| Body mass (BM) | -- |  |  |  |  |  |
| Testes mass (TM) | .332  (24) | -- |  |  |  |  |
| Seminal vesicles mass (SVM) | -.004  (24) | -.182  (24) | -- |  |  |  |
| Preputial glands mass (PGM) | .021  (24) | -.234  (24) | -.058  (24) | -- |  |  |
| Baculum length (BL) | -.118  (22) | .559**  (22) | -.342  (22) | -.124  (22) | -- |  |
| Baculum base width (BBW) | .166  (23) | .304  (23) | -.124  (23) | -.412*  (23) | .224  (22) | -- |
| Baculum shaft width | -.022  (22) | .120  (22) | .029  (22) | -.184  (22) | .370  (22) | .662**  (22) |

**Additional file 1: Table S3** Generalized linear mixed model to investigate morphological traits influencing the reproductive success of male house mice, including PCA for baculum size measurements (PCA1), showing the best model for total number of offspring sired. Data for morphological traits were log transformed prior to analysis. Body mass was retained in the models as a covariate. Population and sibling group were included as random effects (see main text for further details). ΔAIC = the change in the AIC if the single term is dropped. Number of observations = 22, sibling groups = 11, populations = 4.

| **Fixed effects** | **Coefficient (SE)** | **z-value** | ***P*-value** | **ΔAIC** | **Random**  **effects** | **Variance (SD)** |
| --- | --- | --- | --- | --- | --- | --- |
| *Model for total number of offspring sired* |  |  |  |  |  |  |
| Intercept | -21.78  (4.74) | -4.59 | <0.001 | - | Sibling group | 0.58 (0.76) |
| Body mass | 7.73  (1.52) | 5.07 | **<0.001** | - | Population | 0.43 (0.66) |
| Baculum PC1 | 1.10  (0.18) | 6.04 | **<0.001** | 54.22 |  |  |

**Additional file 1: Table S4** Linear mixed model to investigate morphological traits influencing the reproductive success of male house mice, including PCA for baculum size measurements (PCA1), showing the best model for average number of offspring sired per litter. Data for morphological traits were log transformed prior to analysis. Body mass was retained in the model as a covariate. Population and sibling group were included as random effects (see main text for further details). ΔAIC = the change in the AIC if the single term is dropped. Number of observations = 19, sibling groups = 11, populations = 4.

| **Fixed effects** | **Coefficient (SE)** | **z-value** | ***P*-value** | **ΔAIC** | **Random**  **effects** | **Variance (SD)** |
| --- | --- | --- | --- | --- | --- | --- |
| *Model for average number of offspring per litter* |  |  |  |  |  |  |
| Intercept | -16.65 (5.15) | -3.24 |  | - | Sibling group | 1.23 (1.11) |
| Body mass | 6.13 (1.66) | 3.69 | **<0.001** | - | Population | 0.15 (0.39) |
| Baculum PC1 | 0.72 (0.20) | 3.55 | **0.011** | 4.53 |  |  |

**Additional file 1: Table S5 -** Generalized linear mixed models to investigate morphological traits influencing the reproductive success of male house mice, showing models for total number of offspring sired: a) for each morphological trait analysed separately, and b) for significant traits combined (traits in bold were retained in the best model). Data for morphological traits were log transformed prior to analysis. Body mass was retained in each of the models as a covariate. Population and sibling group of focal males were included as random effects (see main text for further details).

| **Fixed effects** | **n** | | **Coefficient**  **(SE)** | **z-value** | ***P*-value** | **Random**  **effects** | **n** | **Variance**  **(SD)** |
| --- | --- | --- | --- | --- | --- | --- | --- | --- |
| a) Models for individual variables: | |  |  |  |  |  |  |  |
| *i) Testes mass* | 24 | |  |  |  |  |  |  |
| Intercept |  | | -16.10  (3.85) | -4.18 | <0.001 | Sibling group | 12 | 0.38  (0.62) |
| Body mass |  | | 4.98  (1.26) | 3.95 | <0.001 | Population | 4 | 0.01  (0.10) |
| Testes mass |  | | 16.80  (5.00) | 3.36 | <0.001 |  |  |  |
|  |  | |  |  |  |  |  |  |
| *ii) Seminal vesicles mass* | 24 | |  |  |  |  |  |  |
| Intercept |  | | -12.86  (4.03) | -3.19 | <0.002 | Sibling group | 12 | 0.41  (0.64) |
| Body mass |  | | 4.17  (1.34) | 3.10 | <0.002 | Population | 4 | 0.00  (0.00) |
| Seminal vesicles mass |  | | 10.32  (2.67) | 3.86 | <0.001 |  |  |  |
|  |  | |  |  |  |  |  |  |
| *iii) Preputial glands mass* | 24 | |  |  |  |  |  |  |
| Intercept |  | | -14.37  (3.86) | -3.73 | <0.001 | Sibling group | 12 | 0.33  (0.57) |
| Body mass |  | | 5.60  (1.23) | 4.53 | <0.001 | Population | 4 | 0.00  (0.00) |
| Preputial glands mass |  | | -9.23  (5.09) | -1.81 | 0.07 |  |  |  |
|  |  | |  |  |  |  |  |  |
| *iv) Baculum length* | 22 | |  |  |  |  |  |  |
| Intercept |  | | -23.91  (5.05) | -4.73 | <0.001 | Sibling group | 11 | 0.40  (0.63) |
| Body mass |  | | 6.05  (1.27) | 4.78 | <0.001 | Population | 4 | 0.00  (0.00) |
| Baculum length |  | | 5.06  (1.89) | 2.68 | <0.008 |  |  |  |
|  |  | |  |  |  |  |  |  |
| *v) Baculum shaft width* | 22 | |  |  |  |  |  |  |
| Intercept |  | | -24.69  (4.94) | -5.00 | <0.001 | Sibling group | 11 | 0.68  (0.83) |
| Body mass |  | | 6.44  (1.47) | 4.37 | <0.001 | Population | 4 | 0.00  (0.00) |
| Baculum shaft width |  | | 23.72  (4.33) | 5.48 | <0.001 |  |  |  |
|  |  | |  |  |  |  |  |  |
| *vi) Baculum base width* | 23 | |  |  |  |  |  |  |
| Intercept |  | | -27.05  (5.24) | -5.16 | <0.001 | Sibling group | 12 | 1.18  (1.10) |
| Body mass |  | | 8.22  (1.58) | 5.20 | <0.001 | Population | 4 | 0.00  (0.00) |
| Baculum base width |  | | 9.27  (2.01) | 4.62 | <0.001 |  |  |  |
| b) Model for combined variables: | |  |  |  |  |  |  |  |
|  | 22 | |  |  |  |  |  |  |
| Intercept |  | | -22.92  (7.15) | -3.21 | <0.002 | Sibling group | 11 | 0.46  (0.67) |
| **Body mass** |  | | **4.68**  **(1.78)** | **2.62** | **<0.009** | Population | 4 | 0.50  (0.70) |
| **Testes mass** |  | | **33.91**  **(11.85)** | **2.86** | **<0.005** |  |  |  |
| **Seminal vesicles mass** |  | | **8.44**  **(3.65)** | **2.31** | **<0.03** |  |  |  |
| Baculum length |  | | -5.35  (3.80) | -1.41 | 0.16 |  |  |  |
| **Baculum shaft width** |  | | **34.75**  **(7.29)** | **4.77** | **<0.001** |  |  |  |
| Baculum base width |  | | 1.94  (2.60) | 0.75 | 0.45 |  |  |  |
|  |  | |  |  |  |  |  |  |

**Additional file 1: Table S6 –** Linear mixed models to investigate morphological traits influencing the reproductive success of male house mice, showing models for average number of offspring sired per litter: a) for each morphological trait analysed separately, and b) for significant traits combined (traits in bold were retained in the best model). Data for morphological traits were log transformed prior to analysis. Body mass was retained in each of the models as a covariate. Population and sibling group of focal males were included as random effects (see main text for further details).

| **Fixed effects** | **n** | | **Estimate**  **(SE)** | ***t* value** | ***P*-value** | **Random**  **effects** | **n** | **Variance**  **(SD)** |
| --- | --- | --- | --- | --- | --- | --- | --- | --- |
| a) Models for individual variables: | |  |  |  |  |  |  |  |
| *i) Testes mass* | 21 | |  |  |  |  |  |  |
| Intercept |  | | -12.22  (7.17) | -1.74 | -- | Sibling group | 12 | 0.38  (0.58) |
| Body mass |  | | 4.15  (2.41) | 1.72 | -- | Population | 4 | 0.49  (0.07) |
| Testes mass |  | | 9.83  (9.27) | 1.06 | 0.30 |  |  |  |
|  |  | |  |  |  |  |  |  |
| *ii) Seminal vesicles mass* | 21 | |  |  |  |  |  |  |
| Intercept |  | | -12.28  (7.47) | -1.64 | -- | Sibling group | 12 | 0.27  (0.52) |
| Body mass |  | | 4.70  (2.46) | 1.91 | -- | Population | 4 | 0.00  (0.00) |
| Seminal vesicles mass |  | | 0.35  (4.63) | 0.08 | 0.94 |  |  |  |
|  |  | |  |  |  |  |  |  |
| *iii) Preputial glands mass* | 21 | |  |  |  |  |  |  |
| Intercept |  | | -11.01  (6.67) | -1.65 | -- | Sibling group | 12 | 0.40  (0.64) |
| Body mass |  | | 4.71  (2.14) | 2.20 | -- | Population | 4 | 0.00  (0.00) |
| Preputial glands mass |  | | -15.32  (7.72) | -1.98 | 0.07 |  |  |  |
|  |  | |  |  |  |  |  |  |
| *iv) Baculum length* | 19 | |  |  |  |  |  |  |
| Intercept |  | | -12.52  (10.52) | -1.19 | -- | Sibling group | 11 | 0.00  (0.00) |
| Body mass |  | | 4.49  (2.64) | 1.70 | -- | Population | 4 | 0.00  (0.00) |
| Baculum length |  | | 0.60  (3.81) | 0.59 | 0.03 |  |  |  |
|  |  | |  |  |  |  |  |  |
| *v) Baculum shaft width* | 19 | |  |  |  |  |  |  |
| Intercept |  | | -17.08  (7.12) | -2.38 | -- | Sibling group | 11 | 0.72  (0.85) |
| Body mass |  | | 5.38  (2.22) | 2.42 | -- | Population | 4 | 0.00  (0.00) |
| Baculum shaft width |  | | 9.50  (6.50) | 1.46 | 0.02 |  |  |  |
|  |  | |  |  |  |  |  |  |
| *vi) Baculum base width* | 20 | |  |  |  |  |  |  |
| Intercept |  | | -21.29  (4.79) | -4.44 | -- | Sibling group | 12 | 1.44  (1.20) |
| Body mass |  | | 6.39  (1.47) | 4.34 | -- | Population | 4 | 0.08  (0.29) |
| Baculum base width |  | | 9.76  (2.22) | 4.39 | 0.03 |  |  |  |
| b) Model for combined variables: | |  |  |  |  |  |  |  |
|  | 19 | |  |  |  |  |  |  |
| Intercept |  | | -29.06  (4.31) | -6.74 | -- | Sibling group | 11 | 1.85  (1.36) |
| **Body mass** |  | | **6.68**  **(0.95)** | **7.06** | **--** | Population | 4 | 0.29  (0.54) |
| Baculum length |  | | 3.15  (1.62) | 1.95 | 0.10 |  |  |  |
| **Baculum shaft width** |  | | **7.95**  **(3.50)** | **2.27** | **0.05** |  |  |  |
| **Baculum base width** |  | | **10.06**  **(1.75)** | **5.76** | **<0.005** |  |  |  |
|  |  | |  |  |  |  |  |  |

**Additional file 1: Table S7 -** Generalized linear mixed model to investigate morphological traits influencing the reproductive success of male house mice, showing the best model for total number of offspring sired, with population added as a fixed effect. Data for morphological traits were log transformed prior to analysis. Body mass was retained in the model as a covariate. Sibling group of focal males was included as a random effect (see text for further details). ΔAIC = the change in the AIC if the single term is dropped. Number of observations = 22, sibling groups = 11.

| **Fixed effects** | **Coefficient (SE)** | **z-value** | ***P*-value** | **ΔAIC** | **Random**  **effects** |  | **Variance (SD)** |  |
| --- | --- | --- | --- | --- | --- | --- | --- | --- |
| *Model for total number of offspring sired* |  |  |  |  |  |  |  |  |
| Intercept | -26.94  (4.96) | -5.43 | <0.001 | - | Sibling group |  | 0.05 (0.22) |  |
| Body mass | 5.04  (1.30) | 3.87 | **<0.001** | - |  |  |  |  |
| Testes mass  Baculum shaft width | 30.10  (5.82)  32.72  (4.85) | 5.82  6.74 | **<0.001**  **<0.001** | 20.81  62.64 |  |  |  |  |
| Population B  Population C  Population D | -1.96  (0.42)  -0.20  (0.36)  -1.53  (0.47) | -4.70  -0.56  -3.26 | <0.001  n.s.  <0.01 | 20.81 |  |  |  |  |
|  |  |  |  |  |  |  |  |  |

**Additional file 1: Table S8 –** Linear mixed model to investigate morphological traits influencing the reproductive success of male house mice, showing the best model for average number of offspring sired per litter, with population added as a fixed effect. Data for morphological traits were log transformed prior to analysis. Body mass was retained in the model as a covariate. Sibling group of focal males was included as a random effect (see text for further details). ΔAIC = the change in the AIC if the single term is dropped. Number of observations = 19, sibling group = 11.

| **Fixed effects** | **Estimate (SE)** | ***t* value** | ***P*-value** | **ΔAIC** | **Random**  **effects** | **Variance**  **(SD)** |
| --- | --- | --- | --- | --- | --- | --- |
|  |  |  |  |  |  |  |
| *Model for average number of offspring per litter* |  |  |  |  |  |  |
| Intercept | -22.22 (3.75) | -5.92 | - | - | Sibling group | 1.30  (1.14) |
| Body mass | 6.10  (1.12) | 5.43 | **<0.001** | - |  |  |
| Baculum shaft width | 9.56  (4.23) | 2.26 | **0.013** | 4.20 |  |  |
| Baculum base width  Population B  Population C  Population D | 8.82  (2.08)  -1.51  (0.99)  0.37  (1.20)  -0.12  (1.19) | 4.24  -1.52  0.31  -0.10 | **0.020**  0.23 | 3.39  1.70 |  |  |

**Additional file Methods**

**Statistical analysis**

*Principal components analysis of baculum size:* We performed a principal components analysis (PCA) of baculum measurements (length, shaft, width, base width) to reduce the number of parameters in our initial analyses. The PCA produced a single principal component (PC) with an eigenvalue greater than 1 (1.84), which explained 61.2% of the variation in baculum size. The loadings of the three baculum measurements on this PC1 were 0.89 (shaft width), 0.83 (base width) and 0.60 (length). The PC score (‘baculum PC1’) was used in initial analyses to explore the influence of baculum size on measures of male reproductive success (Tables S3 and S4). Statistical analyses were conducted using the same approach and procedures described in the main text (see Experimental Procedures – *Statistical analysis*). Further analyses (reported in the main text) were then conducted using separate baculum measurements to explore the relative importance of baculum length and width in explaining variation in male reproductive success.
